# Phytobiotic Supplementation as a Sustainable Alternative to Antibiotic Growth Promoters: Impacts on Growth, Carcass Traits, Blood Lipids and Faecal Microbial Characteristics in Sonali Chickens

**DOI:** 10.1155/vmi/2888910

**Published:** 2026-10-18

**Authors:** Md. Ahsan Habib, Ashikul Islam, Ummay Salma, Md. Nurul Amin, Sabbir Hossen Sabuz, Ayesha Siddika, Md. Al Fattah

**Affiliations:** ^1^ Department of Animal Science and Nutrition Faculty of Veterinary and Animal Science Hajee Mohammad Danesh Science and Technology University Dinajpur 5200 Bangladesh hstu.ac.bd

**Keywords:** antibiotic alternative, growth performance, phytobiotic, serum lipid profile, Sonali chicken

## Abstract

The poultry industry needs safe, residue‐free alternatives to antibiotic growth promoters. Phytobiotics are plant‐derived feed additives that have emerged as promising alternatives, but little is known about how they affect Sonali chickens, a popular dual‐purpose breed in South Asia. This study tested whether the commercial phytobiotic Novovital, given in drinking water, could improve the growth, health and profitability of Sonali birds. A total of 120‐day‐old chicks were divided into four groups and reared for 63 days, receiving 0 (control), 0.5, 1.0 or 1.5 mL of Novovital per litre of drinking water. The 1.0 mL/L dose significantly improved final body weight, feed conversion ratio, breast, thigh and drumstick yield, serum total cholesterol and high‐density lipoprotein (HDL) cholesterol and faecal total viable bacterial count (*p* < 0.05). Birds receiving 1.0 mL/L had approximately 10% greater final body weight than control birds (*p* < 0.05), converted feed more efficiently and produced more breast, thigh and drumstick meat, even though all groups ate the same amount of feed. The same dose lowered blood cholesterol, increased HDL cholesterol and reduced the total viable bacterial count in faeces by approximately 75% relative to the control (*p* < 0.05), while all other blood values remained unchanged. No mortality was observed at the 1.0 and 1.5 mL/L doses, and meat drip loss was lowest at 1.0 mL/L. Numerically, net profit per bird at this dose was more than double that of the control (*p* > 0.05). Increasing the supplementation level to 1.5 mL/L did not confer additional benefits. Overall, Novovital at 1.0 mL/L improved growth, feed efficiency and profitability in Sonali production without adverse effects on bird health.

## 1. Introduction

Among livestock enterprises, poultry ranks as a low‐cost and highly efficient supplier of animal protein, and it is pivotal for narrowing the protein deficit in densely populated, resource‐constrained countries, such as Bangladesh. The Sonali bird, a dual‐purpose cross obtained by mating Rhode Island Red sires with Fayoumi dams, has gained widespread acceptance in Bangladesh, since it pairs robustness and satisfactory meat quality with a taste resembling that of native fowl, which makes it a good fit for both backyard and semi‐intensive operations. With consumer demand for poultry meat climbing steadily, farmers face mounting pressure to raise growth rate and feed efficiency while keeping their birds healthy and the enterprise profitable [1].

Over many years, low‐dose antibiotics were routinely incorporated into poultry rations to secure such improvements. This approach has since been broadly curtailed, since the sustained and indiscriminate reliance on antibiotic additives has promoted the emergence of resistant microbes and deposited residues in edible products, thereby endangering the health of people and livestock alike [2–4]. Consequently, the search for alternatives that are safe, effective and free of residues now ranks among the central problems confronting contemporary animal nutrition.

Among these candidates, phytobiotics (plant‐derived feed additives comprising essential oils, herbs, spices and their derivatives) have emerged as promising alternatives [5–7]. Their bioactive constituents, including phenolics, flavonoids, saponins and terpenes, may improve intestinal morphology, stimulate digestive enzyme secretion, favour beneficial members of the intestinal microbiota and counter the oxidative stress associated with rapid growth [8–11]. These actions translate into measurable production benefits: dietary phytobiotics have enhanced live weight gain and feed conversion in chickens, modulated serum lipid profiles and reduced intestinal pathogen loads [12–16]. Such effects are nonetheless dose‐dependent: intermediate levels generally improve performance, whereas excessive levels may suppress feed consumption and growth, making the determination of an optimal inclusion rate critical for both effectiveness and cost.

Work on Sonali chickens specifically remains limited and has followed a narrow set of approaches. Dietary supplementation with *Asparagus racemosus* and *Withania somnifera* root powders improved weight gain and altered the faecal microbial population and blood parameters [17], and papaya leaf juice has been evaluated for its effects on performance and faecal bacterial load in the same breed. Antimicrobial growth promoters, such as halquinol, have likewise been tested in Sonali birds and improved body weight gain [18]. Two gaps follow: First, almost all published work has delivered the additive through the feed as a ground plant material; delivery of a standardised liquid phytobiotic through the drinking water (the route most accessible to smallholders, who commonly buy compounded feed but medicate their own water) remains largely unexamined in this breed. Second, no study has assessed a single commercial phytobiotic simultaneously across growth, carcass output, serum lipid profile, faecal bacterial load and economic return, so the trade‐off between the cost of supplementation and the return it generates is unknown for Sonali producers.

Accordingly, the present investigation examined how the commercial phytobiotic Novovital, delivered via drinking water at graded concentrations, influences Sonali chickens. The study aimed to quantify the effects of supplementation on growth performance and carcass attributes, on the blood‐biochemical and serum lipid profile and on faecal bacterial counts, and to appraise the economic efficiency of production. Since the trial did not include an antibiotic‐treated positive control, its scope is limited to characterising the response to Novovital relative to unsupplemented birds and to identifying the most effective inclusion rate; direct comparison with conventional antibiotic growth promoters was outside its design.

## 2. Materials and Methods

### 2.1. Ethical Approval

All procedures involving live birds followed the institutional rules governing the care and use of research animals, and the study protocol was approved by the Animal Ethics Committee of the Department of Animal Science and Nutrition, Hajee Mohammad Danesh Science and Technology University (HSTU), Dinajpur, Bangladesh (Approval No. HSTU/VAS/ASN/EA/035). The birds were treated humanely for the entire duration of the trial, and slaughter was carried out by skilled staff in order to reduce stress and discomfort.

### 2.2. Study Location and Experimental Period

The feeding experiment took place at the Central Poultry Farm of HSTU, Dinajpur, Bangladesh, across 63 days spanning May to July 2024. Blood haematology and serum biochemistry assays, together with faecal bacterial counting, were performed at the outside laboratories named in the corresponding sections that follow.

### 2.3. Experimental Birds and Design

One hundred and twenty healthy, disease‐free, day‐old Sonali chicks (dual‐purpose cross of Rhode Island Red and Fayoumi) were procured from local dealers in Dinajpur. Upon arrival, the chicks were brooded as a single group for the initial 7 days to permit acclimatisation and were subsequently randomly assigned to four treatment groups (T0–T3) in a completely randomised design, comprising three replicate pens for each treatment and 10 birds in every pen (30 birds per treatment; 120 birds altogether). Each replicate was handled in the same manner apart from the allotted dietary treatment (Table 1).

**TABLE 1:** Experimental layout and allocation of birds.

Dietary treatment (Novovital dose)	Number of Sonali chickens in each replication			Total
	R1	R2	R3	
T0—control (basal diet; 0 mL/L)	10	10	10	30
T1—basal diet + 0.5 mL/L	10	10	10	30
T2—basal diet + 1.0 mL/L	10	10	10	30
T3—basal diet + 1.5 mL/L	10	10	10	30
Total number of birds	40	40	40	120

*Note:* R1, R2, and R3 denote the three replicate pens per treatment. Novovital (Square Pharmaceuticals Ltd.) was supplied through the drinking water.

### 2.4. Dietary Treatments and Feed Additives

The commercial phytobiotic Novovital (Square Pharmaceuticals Ltd., Bangladesh) is shown in Table 2. Nutrient composition of the basal starter and finisher rations is presented in Table 3) was delivered through the drinking water at four graded concentrations: 0 (T0, control), 0.5 (T1), 1.0 (T2) and 1.5 (T3) mL per litre of water (the dose range was centred on the manufacturer’s recommended inclusion rate of 1.0 mL/L, with a half‐strength level below it and a 1.5‐fold level above it, so that responses below, at and above the commercial recommendation could be compared). Every group was fed an identical basal ration, formulated using maize, rice polish and soyabean meal and compounded to satisfy the nutrient needs of growing Sonali birds. All chicks were reared under common management during the first week (Days 0–7) to allow adaptation to the brooding environment, to complete the routine hatchery and early vaccination schedule and to permit the gut and immune systems to stabilise; water intake during this period is low and irregular, which would have prevented uniform delivery of a water‐soluble additive. Excluding the first week also removed brooding‐related early mortality, which is unrelated to dietary treatment. Feeding proceeded in two phases: a starter ration from Day 8 to Day 20 and a finisher ration from Day 21 until the trial ended (Table 4). Feed and clean drinking water were provided *ad libitum*.

**TABLE 2:** Composition of Novovital liquid.

Component	Chemical name	Carbon chain	Classification
1	Butyric acid monoesters	C4	Short‐chain fatty acid (SCFA) derivative
2	Caprylic acid monoesters	C8	Medium‐chain fatty acid (MCFA) derivative
3	Capric acid monoesters	C10	Medium‐chain fatty acid (MCFA) derivative
4	Lauric acid monoesters	C12	Medium‐chain fatty acid (MCFA) derivative

**TABLE 3:** Nutrient composition of the basal starter and finisher rations.

Nutrient/ingredient	Starter (0–30 days)	Grower (31–63 days)
Metabolisable energy, min (kcal/kg)	2950	3000
Moisture, max (%)	12	12
Crude protein, min (%)	20	19
Crude fat, min (%)	4	4.5
Crude fibre, max (%)	4	4
Lysine, min (%)	1.2	1.1
Methionine, min (%)	0.45	0.40
Calcium, min (%)	1.00	0.90
Phosphorus, min (%)	0.48	0.45
Vitamins and minerals	As per requirement	As per requirement

*Note:* Source: Nahar Poultry and Hatchery Limited, Bangladesh. min = Minimum and max = Maximum.

**TABLE 4:** Composition of the basal starter and finisher rations.

Ingredient	Starter diet (Days 8–20)	Finisher diet (from Day 21)
Maize	58.00	58.00
Rice polish	10.00	10.00
Soyabean meal	21.00	20.00
Soyabean oil	0.50	1.00
Molasses	0.50	0.50
Fish meal	6.50	6.00
Limestone	1.50	2.00
Dicalcium phosphate (DCP)	0.70	1.00
Methionine	0.10	0.12
Lysine	0.05	0.08
Vitamin premix	0.35	0.35
SQ Zyme SME (enzyme)	0.10	0.10
Hemeco Sal dry powder	0.20	0.20
Turbo Tox (toxin binder)	0.10	0.10
Sodium bicarbonate	0.15	0.15
Choline chloride	0.05	0.05
Salt	0.20	0.35
Total	100.00	100.00

*Note:* Amounts are expressed as kilograms per 100 kg of complete feed. Source: Nahar Feed Ltd.

### 2.5. Housing, Management and Vaccination Programme

Birds were housed in a bamboo‐and‐wire‐netting shed divided into 12 pens measuring approximately 1.22 × 1.22 m. Before stocking, the shed and all fittings were scrubbed, washed and sanitised with bleaching powder, kept empty for 2 weeks and treated once more with Timsen solution (1 mL per 3 L of water). A potassium permanganate footbath was placed at the entrance, drinkers were rinsed each day, and feeders were cleaned once a week. Fresh rice‐husk bedding (about 5–7 cm deep) laid over a calcium‐carbonate base was used and topped with paper during brooding; soiled litter was removed regularly, turned three times weekly and disinfected every other day. While brooding, an electrical brooder held the temperature near 35°C in week one, lowered by roughly 1°C daily until ambient temperature was reached, and a 23‐h light to 1‐h dark regimen was maintained throughout. Light intensity was set at approximately 30 lux during the first week of brooding and subsequently reduced to about 20 lux from Day 8 until the end of the trial. To curb transport and heat stress, the chicks were given vitamin C, glucose, electrolytes and water‐soluble vitamins during the early days. Birds were vaccinated against Newcastle disease and infectious bronchitis on Day 4 (live ND clone 30 + IB Massachusetts MA5, eye drop), infectious bursal disease on Days 12 and 17 (live intermediate strain D78, eye drop and drinking water, respectively), Newcastle disease and infectious bronchitis on Day 22 (live ND LaSota + IB H120, drinking water) and Newcastle disease on Day 42 (live ND LaSota, drinking water), following the manufacturer’s instructions.

### 2.6. Growth Performance Measurements

Birds and feed were weighed by replicate, from which the indices below were calculated.

 Body Weight: Body weight was recorded at stocking and thereafter at 7‐day intervals through Day 63.
 Body Weight Gain: Derived per replicate by subtracting the starting weight from the final body weight across the matching interval.
 Feed Intake: Obtained as feed supplied minus feed left over, reported on a per‐bird, per‐replicate basis.
 Feed Conversion Ratio (FCR): Computed by dividing feed consumed (kg) by the weight gained (kg).
 Mortality: Mortality was recorded daily for every treatment and given as a proportion of all birds within the group.

### 2.7. Carcass Traits and Meat Quality Evaluation

At Day 63, two birds from each replicate were randomly selected, weighed and slaughtered, with roughly 2 minutes allowed for full bleeding. The dressed body and its segments (breast muscle, thigh muscle, drumstick, wings, shank, head and skin with feathers), along with the viscera (liver, heart, gizzard and abdominal fat), were dissected and individually weighed.

 Dressing Yield: Taken as the eviscerated carcass weight relative to live weight, stated as a percentage (dressing percentage).
 Drip Loss: Drip loss was determined by the suspension (bag) method of Honikel [19], with minor modification. A 20‐g portion taken from the breast muscle of each carcass was sealed in a thin polyethylene bag, suspended inside a refrigerator (4°C) and reweighed at 24 and 48 h postmortem. Drip loss was expressed as the percentage of the initial sample weight lost during storage.

### 2.8. Blood Collection and Haemato‐Biochemical Analyses

At the end of the trial (Day 63), blood was collected from two birds per replicate pen (6 birds per treatment), selected at random from among the birds whose body weight was closest to the pen mean. Birds were fasted for 8 h before sampling. Approximately 5 mL of blood was drawn from the wing (brachial) vein of each bird using a sterile 3 cc needle and syringe. For haematological analysis, 2 mL was transferred to a tube containing K_2_‐EDTA as anticoagulant and processed within 1 h of collection. The remaining 3 mL was placed in a plain tube without anticoagulant for serum separation. Sampling was carried out in the morning between 7 a.m. and 9 a.m. to minimise diurnal variation.

 Complete Blood Count (CBC): Whole‐blood specimens were run on an automated haematology analyser (Sysmex, Japan) at the Prof. Emdadul Haque Pranisheba Kendra and Veterinary Diagnostic Centre, Thakurgaon, to measure haemoglobin (Hb), red blood cell (RBC) and white blood cell (WBC) counts, packed cell volume (PCV), mean corpuscular volume (MCV) and erythrocyte sedimentation rate (ESR).
 Serum Biochemistry: To obtain serum, samples were allowed to clot for 1 h at ambient temperature (∼25°C) and then spun at 2000 rpm for 15 min; the resulting clear, nonhaemolysed serum was held at −20°C pending analysis. Total cholesterol, triglycerides, high‐density lipoprotein cholesterol (HDL‐C), low‐density lipoprotein cholesterol (LDL‐C) and glucose were quantified on an automated biochemistry analyser (Hitachi 7180, Japan) with commercial reagent kits (Randox, Catalogue number: GF2635) at the Department of Pathology and Parasitology, Faculty of Veterinary and Animal Science, HSTU.

### 2.9. Faecal Sampling and Microbial Enumeration

Fresh droppings were collected from every replicate immediately before treatment began (Day 0) and again on Days 14, 35 and 63. Each sample was placed in a clean, sterile container and transported on ice to Toufique Agro Lab, Rangpur. One gram of faeces was homogenised in 9 mL of sterile diluent and serially diluted 10‐fold; total viable bacteria were enumerated by the standard plate count technique on plate count agar following ISO 4833‐1:2013 [20], and plates yielding 30–300 colonies were counted. Bacterial load was expressed as colony‐forming units per gram of faeces (CFU/g).

### 2.10. Economic Analysis

A cost–benefit analysis was carried out for every treatment using current market rates. Total cost per bird was made up of the day‐old chick price, the overall feed cost (feed intake × unit feed price), the phytobiotic cost at the relevant dose and other production costs for labour, electricity, medication, litter and disinfection. Revenue per bird was estimated from the mean live weight and the prevailing local price per kilogram of live bird, and net profit per bird was calculated as revenue less total cost. All monetary figures were stated in US dollars (USD).

### 2.11. Statistical Analysis

All variables were analysed by one‐way analysis of variance (ANOVA) using the general linear model (GLM) procedure, with dietary treatment as the only fixed effect, according to the model *Y*_*i* *j*_ = *μ* + *T*_*i*_ + *ε*_*i* *j*_, where *Y*_*i* *j*_ is the observed value, *μ* is the overall mean, *T*_*i*_ is the effect of the *i*th treatment (*i* = 1–4), and *ε*_*i* *j*_ is the random residual error. Variables recorded on more than one occasion (body weight, body weight gain, feed intake and FCR at 7‐day intervals and faecal bacterial load on Days 0, 14, 35 and 63) were analysed by a separate one‐way ANOVA at each time point rather than by a repeated‐measures model, and no adjustment was made for testing across successive time points. Carcass and meat quality traits, haematological and serum biochemical variables, mortality (expressed as the pen‐level percentage) and the cost–benefit variables were analysed with the same one‐way ANOVA model.

Where the overall F‐test indicated a significant treatment effect, treatment means were separated post hoc by Duncan’s multiple range test. The level of significance was set a priori at *α* = 0.05 for all analyses; it was specified before the data were collected and was not altered during the analysis. Differences are described as significant at *p* < 0.05 and highly significant at *p* < 0.01, and as nonsignificant (NS) at *p* ≥ 0.05; these are denoted by ^∗^, ^∗∗^ and NS, respectively, in the tables and figures.

All results are presented as means ± standard error of the mean (SEM). All analyses were performed with IBM SPSS Statistics for Windows, Version 25.0 (IBM Corp., Armonk, NY, USA), and the figures were prepared with GraphPad Prism 10.

## 3. Results

The dietary supplementation of the phytobiotic Novovital was evaluated for its effects on the productive performance, carcass traits, haemato‐biochemical profile, faecal microbial load, meat quality and economics of Sonali chickens. The results for each parameter are presented below.

### 3.1. Live Weight

Initial live weight (Day 1) and live weight up to Day 21 did not differ significantly among the treatment groups (*p* > 0.05). From Day 28 onwards, live weight differed significantly among treatment groups (*p* < 0.05). Final live weight at Day 63 was significantly higher in T2 (842.83 ± 0.73 g) than in the control T0 (769.25 ± 0.38 g) (*p* < 0.05), with T3 (815.30 ± 1.19 g) and T1 (777.42 ± 1.31 g) intermediate (Figure 1).

**FIGURE 1:**
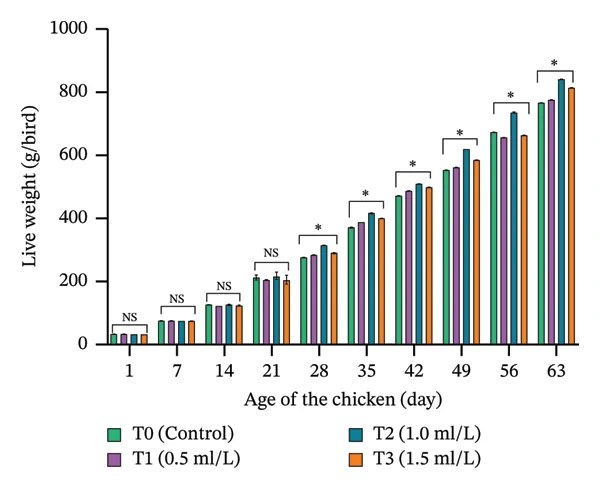
Influence of dietary phytobiotic (Novovital) supplementation on the live weight (g) of Sonali birds. Legends: Data are expressed as means ± SEM. NS, nonsignificant (*p* > 0.05); ^∗^, *p* < 0.05. T0 was the control given the basal diet alone, while T1, T2 and T3 received the basal diet supplemented with Novovital at 0.5, 1.0 and 1.5 mL/L of drinking water, respectively.

### 3.2. Live Weight Gain

Live weight gain, recorded at 7‐day intervals, did not differ among treatment groups up to Day 28 (*p* > 0.05) but became significant from Day 35 to Day 56 and at Day 63 (*p* < 0.05). Over the whole rearing period (0–63 days), the highest total weight gain was achieved by T2 (811.13 ± 0.67 g) and the lowest by the control T0 (737.52 ± 0.56 g); T3 and T1 gained 783.63 ± 1.36 g and 745.61 ± 1.42 g, respectively (Figure 2).

**FIGURE 2:**
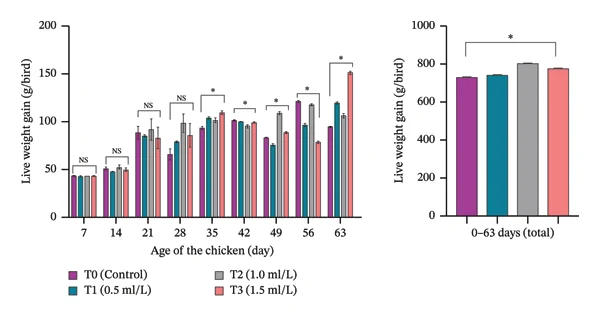
Influence of dietary phytobiotic (Novovital) supplementation on the live weight gain (g) of Sonali birds. Legends: Data are expressed as means ± SEM. NS, nonsignificant (*p* > 0.05); ^∗^, *p* < 0.05. T0 was the control given the basal diet alone, while T1, T2 and T3 received the basal diet supplemented with Novovital at 0.5, 1.0 and 1.5 mL/L of drinking water, respectively.

### 3.3. Feed Intake

Feed intake did not differ significantly among the dietary groups at any time point, nor for cumulative intake over the trial (*p* > 0.05). Total feed intake (1–63 days) was lowest in the control T0 (2015.96 ± 1.13 g) and highest in T1 (2026.87 ± 3.25 g), with T2 and T3 closely comparable (Figure 3).

**FIGURE 3:**
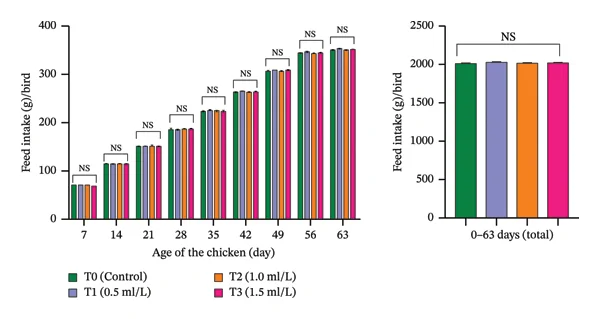
Influence of dietary phytobiotic (Novovital) supplementation on the feed intake (g) of Sonali birds. Legends: Data are expressed as means ± SEM. NS, nonsignificant (*p* > 0.05). T0 was the control given the basal diet alone, while T1, T2 and T3 received the basal diet supplemented with Novovital at 0.5, 1.0 and 1.5 mL/L of drinking water, respectively.

### 3.4. FCR

FCR was NS from Day 7 to Day 28 (*p* > 0.05) but differed significantly from Day 35 to Day 63 (*p* < 0.05). The most favourable (lowest) cumulative FCR was obtained in T2 (2.49 ± 0.00), while T1 had the highest (2.72 ± 0.01); the control T0 and T3 recorded 2.69 ± 0.00 and 2.58 ± 0.00, respectively (Figure 4).

**FIGURE 4:**
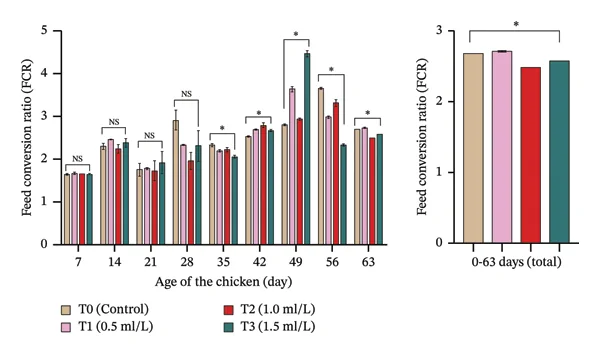
Influence of dietary phytobiotic (Novovital) supplementation on the feed conversion ratio (FCR) of Sonali birds. Legends: Data are expressed as means ± SEM. NS, nonsignificant (*p* > 0.05); ^∗^, *p* < 0.05. T0 was the control given the basal diet alone, while T1, T2 and T3 received the basal diet supplemented with Novovital at 0.5, 1.0 and 1.5 mL/L of drinking water, respectively.

### 3.5. Carcass Yield Characteristics

Live weight and carcass weight differed highly significantly among treatment groups (*p* < 0.01), both being highest in T2 (863.53 ± 13.96 g and 527.35 ± 3.76 g) and lowest in the control T0. Breast, thigh and drumstick weights also increased significantly with phytobiotic supplementation (*p* < 0.05). Dressing percentage, abdominal fat, liver, heart, wings, shank, gizzard, head and skin‐and‐feather weights did not differ significantly among groups (*p* > 0.05) (Table 5).

**TABLE 5:** Influence of dietary phytobiotic (Novovital) supplementation on the carcass traits (g) of Sonali birds.

Parameter	Dietary treatment group				Significance
	T0	T1	T2	T3	
Live weight (g)	769.58 ± 5.79^c^	779.33 ± 5.36^c^	863.53 ± 13.96^a^	833.33 ± 5.24^b^	^∗∗^
Carcass weight (g)	467.86 ± 6.44^c^	481.54 ± 3.09^c^	527.35 ± 3.76^a^	511.22 ± 2.38^b^	^∗∗^
Dressing (%)	60.80 ± 0.77	61.79 ± 0.55	61.09 ± 0.61	61.35 ± 0.47	NS
Breast (g)	102.00 ± 1.53^b^	106.33 ± 1.20^b^	120.00 ± 3.61^a^	116.33 ± 1.86^a^	^∗^
Thigh muscle (g)	70.00 ± 1.15^b^	72.67 ± 0.88^b^	79.00 ± 1.15^a^	76.67 ± 0.88^a^	^∗^
Drumstick (g)	33.67 ± 0.88^b^	35.33 ± 0.88^b^	42.00 ± 1.15^a^	40.67 ± 0.33^a^	^∗^
Abdominal fat (g)	7.33 ± 0.88	9.00 ± 0.58	11.33 ± 0.67	11.67 ± 1.20	NS
Liver (g)	27.02 ± 0.45	28.80 ± 0.59	28.62 ± 0.46	27.66 ± 0.38	NS
Heart (g)	6.67 ± 0.31	6.89 ± 0.28	7.12 ± 0.31	7.23 ± 0.30	NS
Wings (g)	25.70 ± 0.78	26.62 ± 0.88	28.67 ± 0.88	28.00 ± 0.58	NS
Shank (g)	31.67 ± 0.88	32.33 ± 0.88	34.00 ± 0.58	33.33 ± 0.88	NS
Gizzard (g)	29.67 ± 0.48	28.90 ± 1.00	30.00 ± 1.15	30.33 ± 0.88	NS
Head (g)	37.67 ± 1.20	37.90 ± 1.53	39.67 ± 0.88	39.33 ± 0.88	NS
Skin and feather (g)	121.33 ± 3.53	125.00 ± 2.89	146.00 ± 9.54	139.67 ± 6.49	NS

*Note:* Data are expressed as means ± SEM. Within a row, means bearing unlike superscript letters (a–c) differ significantly (*p* < 0.05). NS, nonsignificant (*p* > 0.05). T0 was the control given the basal diet alone, while T1, T2 and T3 received the basal diet supplemented with Novovital at 0.5, 1.0 and 1.5 mL/L of drinking water, respectively.

^∗^ *p* < 0.05.

^∗∗^ *p* < 0.01.

### 3.6. Serum Biochemical Profile

Total cholesterol decreased significantly with phytobiotic supplementation (*p* < 0.05), being highest in T0 (162.66 ± 1.54 mg/dL) and lowest in T2 (147.01 ± 3.90 mg/dL), while HDL increased significantly, peaking in T2 (55.35 ± 0.85 mg/dL). Serum glucose declined significantly, being lowest in T3 (182.87 ± 9.78 mg/dL). Triglyceride, LDL, Hb, ESR, RBC, PCV, MCV and WBC count did not differ significantly among treatment groups (*p* > 0.05) (Table 6).

**TABLE 6:** Influence of dietary phytobiotic (Novovital) supplementation on the haemato‐biochemical profile of Sonali birds.

Parameter	Dietary treatment group				Significance
	T0	T1	T2	T3	
Total cholesterol (mg/dL)	162.66 ± 1.54^a^	158.11 ± 4.47^a^	147.01 ± 3.90^b^	159.87 ± 1.25^a^	^∗^
Triglyceride (mg/dL)	82.53 ± 5.20	79.03 ± 5.47	64.84 ± 3.44	71.45 ± 2.56	NS
HDL (mg/dL)	39.55 ± 0.41^b^	38.97 ± 1.89^b^	55.35 ± 0.85^a^	52.47 ± 0.97^a^	^∗^
LDL (mg/dL)	81.05 ± 14.34	73.26 ± 3.06	64.48 ± 2.03	66.37 ± 3.35	NS
Hb (g/dL)	10.73 ± 1.53	11.43 ± 0.54	11.53 ± 0.61	11.97 ± 0.23	NS
ESR (mm/1st h)	1.67 ± 0.18	1.30 ± 0.16	1.49 ± 0.11	1.31 ± 0.17	NS
RBC (× 10^6^/μL)	2.56 ± 0.05	2.68 ± 0.10	2.57 ± 0.11	2.67 ± 0.07	NS
PCV (%)	29.99 ± 1.33	28.60 ± 1.65	31.65 ± 1.63	28.81 ± 1.57	NS
MCV (fL)	139.70 ± 1.01	137.83 ± 1.17	139.62 ± 0.54	134.24 ± 2.89	NS
WBC (× 10^6^/mm^3^)	2.83 ± 0.43	2.75 ± 0.08	2.63 ± 0.03	2.57 ± 0.05	NS
Glucose (mg/dL)	233.67 ± 3.99^a^	228.02 ± 3.59^a^	214.04 ± 9.32^a^	182.87 ± 9.78^b^	^∗^

*Note:* Data are expressed as means ± SEM. Within a row, means bearing unlike superscript letters (a, b) differ significantly (*p* < 0.05). NS, nonsignificant (*p* > 0.05). T0 was the control given the basal diet alone, while T1, T2 and T3 received the basal diet supplemented with Novovital at 0.5, 1.0 and 1.5 mL/L of drinking water, respectively.

^∗^ *p* < 0.05.

### 3.7. Faecal Bacterial Load Count

Baseline (Day 0) microbial loads were similar across groups (*p* > 0.05). From Day 14 onwards, the faecal bacterial load differed significantly (*p* < 0.05), declining markedly in all phytobiotic groups relative to the control. On Day 63, the control T0 had the highest load (232.67 ± 9.70), whereas T2 had the lowest (66.33 ± 4.48), followed by T1 (94.33 ± 11.29) and T3 (105.00 ± 2.89) (Figure 5).

**FIGURE 5:**
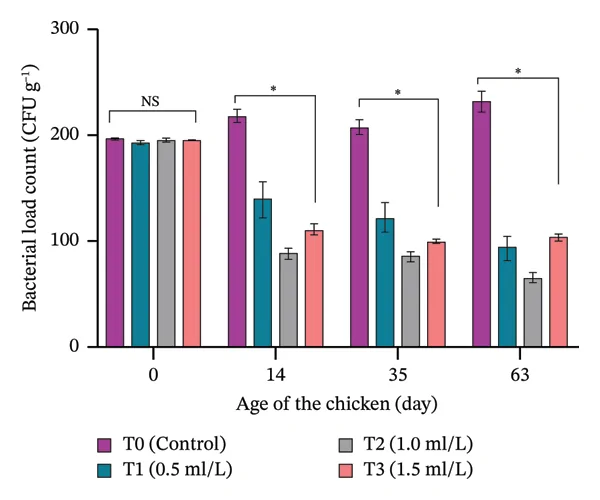
Influence of dietary phytobiotic (Novovital) supplementation on the faecal bacterial load of Sonali birds. Legends: Data are expressed as means ± SEM. Bacterial load is given as the colony count of the faecal sample. NS, nonsignificant (*p* > 0.05); ^∗^, *p* < 0.05. T0 was the control given the basal diet alone, while T1, T2 and T3 received the basal diet supplemented with Novovital at 0.5, 1.0 and 1.5 mL/L of drinking water, respectively.

### 3.8. Drip Loss of Meat and Mortality

Drip loss (24 h) and mortality did not differ significantly among groups (*p* > 0.05). Drip loss was highest in the control T0 (1.47 ± 0.15) and lowest in T2 (1.13 ± 0.09). Mortality was numerically highest in T0 (0.67 ± 0.33%), while no mortality was recorded in either T2 or T3 (Table 7).

**TABLE 7:** Influence of dietary phytobiotic (Novovital) supplementation on drip loss and mortality in Sonali birds.

Parameter	Dietary treatment group				Significance
	T0	T1	T2	T3	
Drip loss (24 h, %)	1.47 ± 0.15	1.20 ± 0.12	1.13 ± 0.09	1.23 ± 0.13	NS
Mortality (%)	0.67 ± 0.33	0.33 ± 0.33	0.00 ± 0.00	0.00 ± 0.00	NS

*Note:* Data are expressed as means ± SEM. NS, nonsignificant (*p* > 0.05). T0 was the control given the basal diet alone, while T1, T2 and T3 received the basal diet supplemented with Novovital at 0.5, 1.0 and 1.5 mL/L of drinking water, respectively.

### 3.9. Cost‐Effectiveness of Sonali Production

Total production cost per bird differed highly significantly (*p* < 0.01), rising with phytobiotic dose from USD 1.719 ± 0.002 (T1) to USD 1.786 ± 0.002 (T3). Average feed consumption, total feed cost, average live weight and sale price per bird also differed significantly (*p* < 0.05). Although net profit per bird was statistically NS (*p* > 0.05), it was numerically highest in T2 (USD 0.293 ± 0.022), followed by T3 (USD 0.208 ± 0.058) and T1 (USD 0.176 ± 0.006), and lowest in the control T0 (USD 0.133 ± 0.014) (Table 8).

**TABLE 8:** Influence of dietary phytobiotic (Novovital) supplementation on the cost–benefit of Sonali production (values in USD).

Parameter	Dietary treatment group				Significance
	T0	T1	T2	T3	
Cost/chick (USD)	0.285 ± 0.000	0.285 ± 0.000	0.285 ± 0.000	0.285 ± 0.000	NS
Feed consumption (kg/bird)	2.09 ± 0.01^a^	2.02 ± 0.00^b^	2.03 ± 0.00^b^	2.03 ± 0.00^b^	^∗^
Feed price (USD/kg)	0.520 ± 0.000	0.520 ± 0.000	0.520 ± 0.000	0.520 ± 0.000	NS
Novovital® cost (USD/bird)	0.000 ± 0.000	0.033 ± 0.000	0.065 ± 0.000	0.098 ± 0.000	NS
Total feed cost (USD/bird)	1.083 ± 0.004^a^	1.052 ± 0.002^b^	1.055 ± 0.002^b^	1.054 ± 0.002^b^	^∗^
Miscellaneous (USD/bird)	0.350 ± 0.000	0.350 ± 0.000	0.350 ± 0.000	0.350 ± 0.000	NS
Total cost/bird (USD)	1.720 ± 0.004^d^	1.719 ± 0.002^c^	1.754 ± 0.002^b^	1.786 ± 0.002^a^	^∗∗^
Avg. live weight (kg)	0.76 ± 0.01^c^	0.78 ± 0.00^bc^	0.84 ± 0.01^a^	0.82 ± 0.02^ab^	^∗^
Sale price (USD/kg)	2.439 ± 0.000	2.439 ± 0.000	2.439 ± 0.000	2.439 ± 0.000	NS
Sale price/bird (USD)	1.853 ± 0.014^c^	1.895 ± 0.007^bc^	2.048 ± 0.024^a^	1.993 ± 0.058^ab^	^∗^
Net profit/bird (USD)	0.133 ± 0.014^ab^	0.176 ± 0.006^b^	0.293 ± 0.022^a^	0.208 ± 0.058^ab^	NS

*Note:* Data are expressed as means ± SEM. Within a row, means bearing unlike superscript letters (a–d) differ significantly (*p* < 0.05). NS, nonsignificant (*p* > 0.05). All monetary values were converted to US dollars at 1 USD = 108 BDT, the Bangladesh Bank mid‐rate on May 2024. T0 was the control given the basal diet alone, while T1, T2 and T3 received the basal diet supplemented with Novovital at 0.5, 1.0 and 1.5 mL/L of drinking water, respectively.

^∗^ *p* < 0.05.

^∗∗^ *p* < 0.01.

## 4. Discussion

### 4.1. Discussion

Novovital improved growth, and the magnitude of the effect depended on dose. Until Day 21, the groups had similar live weights. From Day 28 onwards, significant differences emerged (*p* < 0.05), and by Day 63, the 1.0 mL/L group (T2) was the heaviest, while the control was the lightest, a difference of approximately 10%. The delayed emergence of the treatment effect is consistent with the proposal that the bioactive constituents of phytobiotics exert their greatest influence once the gastrointestinal tract is sufficiently developed to translate enhanced enzyme secretion into improved nutrient uptake [9]. Murugesan et al. [8] attributed comparable gains to increased villus height and reduced crypt depth, which enlarge the absorptive surface available for nutrient uptake. Neither intestinal morphology nor digestive enzyme activity was assessed in the present study, so this mechanism is offered as a hypothesis drawn from previous work rather than as an explanation established here.

Total weight gain followed the same temporal pattern. Gains were similar across groups until Day 28 and then diverged, with T2 gaining the most over the whole rearing period and the control the least. This larger gain with supplementation is consistent with Basit et al. [13], whose phytobiotics equalled or exceeded standard antibiotic growth promoters in broilers. Because the present trial included no antibiotic‐treated positive control, however, no comparison with conventional growth promoters can be drawn from the current data.

Feed intake did not differ among the dietary groups at any time point or over the whole trial (*p* > 0.05). Because all groups consumed comparable amounts of feed, the additional weight gain in supplemented birds cannot be attributed to increased intake and instead indicates improved feed utilisation efficiency.

The FCR supports this interpretation. Cumulative FCR differed significantly among groups (*p* < 0.05), being most favourable in T2 (2.49 ± 0.00) and least favourable in T1 (2.72 ± 0.01), with T3 (2.58 ± 0.00) intermediate and the control T0 at 2.69 ± 0.00. A lower FCR at comparable feed intake indicates improved digestion and nutrient absorption rather than increased consumption. This agrees with Krauze et al. [14], who reported better efficiency without higher intake in broilers given a cinnamon oil phytobiotic, and with Almahallawi et al. [21], who observed improved growth performance in broilers supplemented with *Linum usitatissimum* essential oil. The response in T1 suggests that 0.5 mL/L was insufficient to produce a consistent effect.

These gains were also reflected in carcass yield. Live weight, carcass weight and the breast, thigh and drumstick cuts were significantly higher in T2 (*p* < 0.05), whereas dressing percentage, abdominal fat and organ weights did not differ among groups (*p* > 0.05). The increase in carcass and cut weights without a corresponding increase in abdominal fat or organ weights is consistent with greater deposition of muscle tissue; carcass chemical composition was not determined, however, so this interpretation remains provisional. Comparable increases in carcass and breast yield have been reported by Tavangar et al. [15], Vlaicu et al. [22], Zhang et al. [23] and Yu et al. [24]. The absence of differences in organ weights gives no indication of adverse effects on the organs examined, although a full assessment of metabolic safety would require measures beyond those recorded in this study.

Supplementation was associated with significant changes in the serum lipid profile. Total cholesterol decreased and HDL cholesterol increased, both most markedly in T2 (*p* < 0.05), while serum glucose was lowest in T3 (*p* < 0.05). These changes are consistent with Krauze et al. [14] and with the review of Obianwuna et al. [9]. One mechanism proposed for such effects is inhibition of hepatic HMG‐CoA reductase, the rate‐limiting enzyme in cholesterol biosynthesis, by phenolic and flavonoid compounds; this pathway has been reviewed for dietary flavonoids in poultry by Tan et al. [25]. Enzyme activity was not measured here, and the declared composition of Novovital, so this pathway is presented as a hypothesis supported by previous literature rather than as a mechanism demonstrated in the present birds. Triglyceride, LDL and all haematological parameters did not differ among treatments (*p* > 0.05), and the values recorded were comparable to those reported for healthy chickens [26, 27], although breed‐specific reference ranges are not established for Sonali birds.

One of the clearest results was the reduction in faecal bacterial load. Counts were comparable across groups at baseline, but from Day 14 onwards, the load was significantly lower in all supplemented groups than in the control (*p* < 0.05). By Day 63, T2 carried the lowest load, approximately one‐quarter that of the control. This antibacterial effect agrees with the lower pathogen counts reported by Basit et al. [13], with the reduced *Escherichia coli* and elevated *Lactobacillus* levels described by Paredes‐Lopez et al. [28], and with Yu et al. [24], who reported improved gut health alongside enhanced growth performance and carcass characteristics in broilers given a blend of essential oils. It is also consistent with the mechanism outlined by Burt [29] and Murugesan et al. [8]: essential oil compounds disrupt bacterial cell membranes and lower intestinal pH, suppressing harmful organisms while leaving beneficial populations largely intact. This shift in the gut microbial community likely contributes to the improved growth and feed efficiency observed here.

Drip loss and mortality did not differ significantly among groups (*p* > 0.05). Drip loss was numerically lowest in T2, and mortality was numerically highest in T0 with no deaths recorded in T2 or T3. Because neither difference reached statistical significance, both should be regarded as numerical trends rather than treatment effects; with 30 birds per treatment distributed across three replicate pens, a difference of one bird cannot support inference about survivability. Lower drip loss has been associated with improved water‐holding capacity in broilers given an essential oil blend in the drinking water [30], and antioxidant activity has been proposed as a contributing mechanism [9, 10]. Antioxidant status and meat water‐holding capacity were not measured in the present study.

Total production cost per bird increased with supplementation dose (*p* < 0.01), while average live weight and sale price per bird were significantly higher in T2 than in the control (*p* < 0.05). Net profit per bird was numerically greatest in T2 but did not differ significantly among treatments (*p* > 0.05). Because the pen served as the experimental unit and each treatment comprised only three replicate pens, the statistical power available for this comparison was limited. The apparent economic advantage of T2 should therefore be interpreted as a numerical trend requiring confirmation in a larger trial rather than as a demonstrated difference in profitability.

Several limitations should be considered when interpreting these findings. Water intake was not recorded, so the actual per‐bird intake of the phytobiotic could not be verified and the dose–response relationship rests on the nominal water concentration. Only total viable bacteria were enumerated, which cannot distinguish suppression of pathogenic from beneficial populations, and intestinal histomorphology, digesta pH, digestive enzyme activity and antioxidant status were not assessed; the mechanisms proposed above therefore remain hypotheses derived from previous literature. Birds were unsexed and the sex ratio within pens was not determined, so differences in sex distribution between pens cannot be excluded as a source of variation in body weight and carcass traits. No antibiotic‐treated positive control was included, so the study characterises the response to Novovital relative to supplemented birds and cannot establish equivalence or superiority to conventional antibiotic growth promoters.

Overall, 1.0 mL/L Novovital (T2) produced the most favourable response, with significantly greater live weight, feed conversion efficiency, carcass and cut weights, serum HDL cholesterol and reduced total cholesterol and faecal total viable bacterial count relative to the control (*p* < 0.05), and no adverse effects on the parameters measured. The response was not dose‐dependent beyond 1.0 mL/L: performance at 1.5 mL/L (T3) was weaker than at 1.0 mL/L, matching the reduced benefit at higher inclusion levels reported by Behboodi et al. [31] for a phytogenic blend given in the drinking water. Taken together, these findings indicate that Novovital at 1.0 mL/L improves productive performance, carcass yield, serum lipid profile and faecal bacterial load in Sonali chickens under the conditions tested. Whether it can substitute for antibiotic growth promoters requires direct comparison against an antibiotic‐treated positive control.

## 5. Conclusion

The present study showed that supplementing the drinking water of Sonali chickens with the phytobiotic Novovital improved productive performance, carcass yield and the serum lipid profile, and reduced the faecal total viable bacterial count, relative to unsupplemented birds. The 1.0 mL/L dose (T2) produced the most favourable overall responses, giving the heaviest birds, the most efficient feed conversion, greater yields of the major carcass cuts, reduced serum total cholesterol alongside elevated HDL cholesterol and the lowest faecal bacterial load (*p* < 0.05). Net profit per bird was numerically highest at this dose but did not differ significantly among treatments (*p* > 0.05) and should therefore be regarded as a trend requiring confirmation. No adverse effects were observed in the parameters measured, although welfare indicators, intestinal morphology and microbial composition were not assessed, so no conclusion can be drawn regarding faecal microbial characteristics or bird welfare. The response was not dose‐dependent beyond 1.0 mL/L, indicating that benefits do not increase continuously with concentration. Under the conditions of this study, Novovital at 1.0 mL/L can be recommended as an effective and economically viable supplement for Sonali production; however, because no antibiotic‐treated positive control was included, its efficacy relative to conventional antibiotic growth promoters remains to be established and warrants direct comparison in future work.

## Author Contributions

Md. Ahsan Habib: conceptualisations, methodology, supervision, validation, funding acquisition, writing–original draft, project administration, writing–review and editing, software, resources, data curation, formal analysis and investigation; Ashikul Islam: data collection, data analysis, writing–original draft, data curation, formal analysis, investigation and methodology; Ummay Salma: supervision, validation and ethical approval; Md. Nurul Amin: investigation, validation and data analysis; Sabbir Hossen Sabuz: supervision, investigation and validation; Ayesha Siddika: data analysis and resources; Md. Al Fattah: writing–review and editing, formal analysis, visualisation, software and resources.

## Funding

This study was funded by Special Research Grant 2023‐2024 (Biological Science Group), Ministry of Science and Technology (MoST), The Government of the People’s Republic of Bangladesh, Memo No: 39.00.0000.009.99.023.23‐363, Date: Dec 18, 2023 (Serial No: 204, Project ID: SRG‐231204).

## Disclosure

All authors have read and approved the final version of the manuscript. The corresponding author (or manuscript guarantor) had full access to all of the data in this study and takes complete responsibility for the integrity of the data and the accuracy of the data analysis. The corresponding author affirms that this manuscript is an honest, accurate and transparent account of the study being reported; that no important aspects of the study have been omitted; and that any discrepancies from the study as planned (and, if relevant, registered) have been explained. The funders had no role in the study design, data collection, analysis or interpretation. They also had no role in the decision to submit the article for publication to a specific journal.

## Conflicts of Interest

The authors declare no conflicts of interest.

## Data Availability

The data that support the findings of this study are available on request from the corresponding author. The data are not publicly available due to privacy or ethical restrictions.
